# Marriage and Malthus

**Published:** 1913-11-22

**Authors:** Binnie Dunlop

**Affiliations:** Alexandra Court, S.W.


*To the Editor of* The Hospital.

### Marriage and Malthus.

*To the Editor of* The Hospital.

Sir,—In your Note recording the formation of a Commission of Inquiry which the National Council of Public Morals has established on the declining birth-rate you remark that "the composition of this privately formed body is somewhat curious," inasmuch as "the clergy and bishops are well to the fore," though "the question is a scientific rather than a moral one." Now I am very glad the Church *is* strongly represented. For this question does happen to be regarded as largely a moral one, and a declaration from the Commission that marriage must now be looked upon as primarily for companionship and only secondarily for the begetting of children would enable doctors, hospitals, district nurses, and other social workers to make a speedy end of poverty and a rapid reduction of prostitution and venereal disease. I know that many medical practitioners are only hanging back from advancing the Neo-Malthusian doctrine of early marriage and small families in deference to the attitude of the leaders of official public opinion.—I am, Sir, etc.,

Binnie Dunlop, M.B., Ch.B.

Alexandra Court, S.W.

